# Being Heard, Being Valued, Being Understood—Aboriginal Community Perspectives on Adapting a Healthy Lifestyle Program for Boorloo/Perth, Western Australia: A Qualitative Study

**DOI:** 10.1111/hex.70754

**Published:** 2026-07-16

**Authors:** Stephen Paull, Stephanie Smith, Jordan Bill, Joanna C. Moullin, Tania Harris, Yvonne C. Anderson

**Affiliations:** ^1^ Curtin Medical School, Faculty of Health Sciences Curtin University Bentley Western Australia Australia; ^2^ Child and Adolescent Community Health, Child and Adolescent Health Service Perth Western Australia Australia; ^3^ The Kids Research Institute Australia, Perth Children's Hospital Nedlands Western Australia Australia; ^4^ School of Population Health, Faculty of Health Sciences Curtin University Bentley Western Australia Australia; ^5^ Health Consumers' Council WA Mount Lawley Western Australia Australia; ^6^ Department of Paediatrics: Child and Youth Health, Faculty of Medical and Health Sciences University of Auckland Grafton Auckland New Zealand

**Keywords:** Australian Aboriginal and Torres Strait Islander Peoples, child health, health promotion, healthy lifestyle, implementation science, paediatric obesity, public health, qualitative

## Abstract

**Background:**

Childhood obesity prevalence is increasing globally, with Aboriginal and Torres Strait Islander children over‐represented in Australian data. Evidence‐based, community healthy lifestyle programs require culturally safe adaptation when implemented in new First Nations contexts, where prioritising access and engagement of First Nations groups is critical. This study aimed to identify Aboriginal community representatives' perspectives on potential barriers and enablers to recipient engagement and program implementation, and to identify cultural and place‐based considerations to inform culturally safe adaptation of Whānau Pakari from Aotearoa/New Zealand for delivery as the Healthy Lifestyle Program in Boorloo/Perth, Western Australia.

**Methods:**

A 3‐h workshop was conducted with 29 Aboriginal advisors, with whole‐group discussions and 5 facilitated breakout groups, to obtain Aboriginal community guidance on cultural and place‐based considerations needed to adapt the program locally. Data were analysed by Framework Analysis using the updated Consolidated Framework for Implementation Research (CFIR) to identify barriers and enablers (determinants) of program engagement and implementation. Determinants were then reclassified inductively into themes to enhance participant relatability, supporting communication and feedback.

**Results:**

A total of 44 potential determinants (16 barriers and 28 enablers) were mapped to 18 CFIR constructs across all five domains of innovation, outer setting, inner setting, individuals and implementation process. Three themes were identified from these determinants: *acknowledging cultural context* encompassed healing and self‐determination, reclaiming knowledge and culture, and addressing limited access to health‐promoting environments; *guiding values* included mutual respect, building and earning trust, and intergenerational learning; and *program considerations* included culturally secure practice, specific ways of working, and keeping families engaged.

**Conclusions:**

Aboriginal community guidance highlighted cultural and place‐based priorities for program adaptation, and potential determinants of successful implementation within a prevailing healthcare service, iteratively informing program adaptation. This study used a unique methodological approach, prioritising participant voice alongside CFIR terminology, to provide rare evidence of pre‐implementation First Nations engagement outcomes when adapting a program to integrate into the prevailing healthcare service. Partnership with First Nations communities is essential to implementing accessible, culturally safe models of care, thereby addressing health inequities.

**Lived Experience or Public Contribution:**

An established Cultural Advisory Group of 12 Aboriginal Elders worked in partnership with the Healthy Lifestyle Program, 10 of whom reviewed the identified themes and sub‐themes throughout the analysis process. Two members were nominated by the group and reviewed the final draft manuscript. Identified determinants iteratively informed program adaptation, guided by the Cultural Advisory Group. This work was also supported and guided by a representative consumer body, Health Consumers' Council WA.

## 1 Background

We are here to do stuff for our friends, family, community. That's what we want to do. And how you bring it together later on, that's up to you guys [the program staff and research team] down the track. But we don't want to be cut out of doing stuff for our mob.

Whole‐group discussion (Elder, Female)

### 1.1 Childhood Obesity and Inequity

The prevalence of childhood obesity and weight‐related comorbidities continues to increase globally. Pooled analysis of over 2000 population‐based measurement studies shows the global prevalence of childhood obesity has increased eightfold from 1975 to 2016 [1]. Within Australia, approximately 38% of children and young people aged 5–19 years were living with overweight or obesity in 2020, a figure expected to increase to 46% by 2035 [2]. This increase has been mirrored by a rise in weight‐related comorbidities [2, 3, 4], increasing the risk of all‐cause mortality in adulthood [5, 6]. The reasons for this increase are multifactorial, including social and economic disadvantage, food environments, reduced opportunities for physical activity, health service access, stigma, and broader structural determinants of health [7, 8, 9]. Children and young people identifying as Aboriginal and Torres Strait Islander are over‐represented in obesity and type 2 diabetes prevalence data [10, 11, 12]. Obesity also has economic and health‐system implications, costing the Australian community $11.8 billion in 2018 [9], while overweight including obesity was the third leading risk factor contributing to disease burden among Aboriginal and Torres Strait Islander people in 2018 [13]. These trends highlight the need for accessible, evidence‐based paediatric healthy lifestyle interventions. Such interventions are recommended to be family‐ and community‐based, multicomponent and multidisciplinary, with at least 26 h of contact and support, incorporating nutrition, physical activity and behavioural and psychosocial support [8, 9, 14, 15, 16]. These recommendations provide the rationale for considering whether an evidence‐informed community‐based model could be adapted for local implementation.

### 1.2 Australian and West Australian Contexts

Australia has a mixed public–private health system, with Medicare funding universal health care. The Commonwealth (national government) is responsible for funding Medicare and supporting primary healthcare services, while the state and territory governments manage public hospitals and most community and mental health services [17].

The Aboriginal and Torres Strait Islander resident population in Western Australia (WA) was approximately 120,000 in 2021, 4.4% of the state's population, compared with 3.8% nationally [18]. Aboriginal and Torres Strait Islander people live in major cities (46%), inner–outer regional WA (21%) and remote–very remote WA (33%) [18]. Boorloo/Perth (henceforth referred to as Perth) is the capital city of WA and has a total general population of almost 2.4 million people [19].

For Aboriginal and Torres Strait Islander people, engagement with health services occurs in the context of a complex history including colonisation, dispossession, racism and forced child removal (referred to as the Stolen Generations) [20]. This has contributed to unresolved intergenerational trauma, with ongoing impacts on social and emotional wellbeing [20], and mistrust in health services. Protective factors include strong connections to family, culture and Country. Health interventions should include trauma‐informed and healing‐aware models, developed locally with community governance [20].

The guiding principles of the WA Aboriginal Health and Wellbeing Framework 2015–2030 outline critical components of contemporary health services required to improve Aboriginal health and wellbeing and close the gap on life expectancy [21]. These include the need for Aboriginal community control and engagement in decision‐making, partnerships with the Aboriginal community in the development of health services, and health services to be accessible to Aboriginal people [21]. These principles align with the United Nations Declaration on the Rights of Indigenous Peoples, which affirms self‐determination and the participation of First Nations peoples in decision‐making, partnership in developing health programs, equitable access to health services and state obligations to take steps to provide the highest attainable standard of health and address the drivers of inequity [22].

### 1.3 Service Gap and Rationale for Adaptation

A 2019 audit of Australian multidisciplinary weight management services found that the availability and accessibility of services for children and young people living with obesity were insufficient for demand, especially for those affected by severe obesity [23]. Existing Perth services provide important care but have limitations, including age or severity‐based eligibility restrictions, geographic limitations (most care provided in hospital outpatient settings), insufficient contact time, absence of health screening in some services, limited local effectiveness data, cost barriers for some families, and an over 2‐year waitlist for the tertiary hospital‐based service [24, 25, 26, 27, 28]. Furthermore, there is a paucity of evidence within existing services' engagement with Aboriginal or Torres Strait Islander communities prior to development or adaptation. These limitations highlight the need for an accessible, community‐based model that can provide health screening and multidisciplinary lifestyle support closer to home, while being adapted with local Aboriginal community guidance and place‐based considerations. To accelerate service availability and delivery, scale‐out and adaptation of existing evidence‐based interventions is recommended over piloting new, untested interventions de novo, provided local place‐based and cultural considerations are honoured [29, 30].

### 1.4 Community Involvement and First Nations Program Adaptation

Consumer and community involvement is essential in health research, including in Australia, with consumers and community members acting as partners in research planning, implementation, evaluation and translation [31]. Consumers can be people with lived experience of a health issue or people who represent the views and interests of a consumer organisation or a community [31]. Consumer and community involvement principles align with Aboriginal community partnership and engagement in health‐service decision‐making, making local community guidance key to culturally safe adaptation of programs intended to benefit Aboriginal people and communities [21].

‘Tribal Turning Point’ is the closest identified example of a childhood lifestyle intervention program adapted for First Nations communities in Australia. It was originally developed with American First Nations communities as a diabetes prevention program, then adapted through participatory action research with a Central Australian Aboriginal community for local Aboriginal children, and delivered by an Aboriginal community‐controlled health organisation [32]. Tribal Turning Point focused primarily on diabetes prevention, while also addressing obesity risk and healthy lifestyle behaviours. To the best of our knowledge, there is limited literature detailing pre‐implementation First Nations engagement outcomes on adapting health interventions for local use by First Nations and non‐First Nations recipients together within the prevailing healthcare service.

### 1.5 Program Selected for Adaptation

To complement existing services in Perth, a community‐based intervention, Whānau Pakari, was funded for adaptation and piloting in East Metropolitan Perth as the ‘Healthy Lifestyle Program’. Whānau Pakari is an effective and equitable community‐based healthy lifestyle program for children and young people living with obesity within Taranaki, a mixed urban–rural region of Aotearoa/New Zealand (henceforth referred to as NZ) [33, 34, 35]. It was deemed scalable, in part due to its equity focus and emphasis on community engagement [33, 36, 37]. ‘Whānau Pakari’ means ‘healthy, self‐assured families that are fully active’ [34]. The program consists of 6 months of weekly community‐based family group sessions, along with home‐based weight‐related health assessments at entry and 6 and 12 months, offering a method of screening for weight‐related comorbidities within a community‐based multicomponent lifestyle education program and bringing healthcare closer to home without compromising quality of care [33].

Whānau Pakari demonstrated high engagement from Māori [34], the First Nations peoples of NZ, and was described by Māori recipients as culturally acceptable, with respectful, compassionate care supporting engagement and partially mitigating the effects of systemic racism on families' willingness to participate [38]. Given these features and the limited availability of directly comparable paediatric healthy lifestyle models prioritising marginalised groups, Whānau Pakari was purposively selected as a plausible evidence‐informed model for local adaptation. Selection was also informed by existing implementation and clinical expertise within the research team, delegating site visits to the NZ‐based program to review suitability for the Perth context, early community engagement, and an opportunity for local adaptation and scale‐out [37].

Although Australia and NZ both have publicly funded health systems and formal obligations/frameworks to address First Nations health inequities [39, 40, 41], Māori and Aboriginal Australian contexts are culturally and historically distinct. Therefore, a program that was acceptable and engaging for Māori could not be assumed to be culturally acceptable and engaging for Aboriginal and Torres Strait Islander families, children and young people in Perth. Local Aboriginal involvement was needed to identify cultural and place‐based considerations for adaptation and ensure cultural safety.

The program pilot was funded by and embedded within Child and Adolescent Community Health (CACH), Child and Adolescent Health Service—the publicly funded health service for children and young people in Perth. The state‐funded East Metropolitan Health Service (EMHS), where this study took place, has a catchment boundary in Perth where 3.8% of residents identify as Aboriginal and/or Torres Strait Islander [42]. However, the over‐representation of Aboriginal and Torres Strait Islander people with obesity and type 2 diabetes in prevalence data, alongside state and national equity commitments, made culturally safe adaptation a priority for program planning [10, 11, 12, 21, 40]. The program's adaptation, implementation and research were undertaken in partnership with Curtin University and The Kids Research Institute Australia, with full protocol and rationale reported elsewhere [37]. In brief, the broader research program involves adapting, implementing and evaluating the Healthy Lifestyle Program in Perth using a multiple‐methods hybrid type II effectiveness‐implementation design. The protocol includes three studies: developing the adapted program with key partners and Aboriginal and Torres Strait Islander advisors for scale‐out; assessing acceptability, appropriateness, feasibility and local clinical outcomes of the adapted pilot program; and assessing program scalability post‐pilot [37].

### 1.6 Current Study

This paper reports Aboriginal community perspectives collected during the pre‐implementation phase of the Healthy Lifestyle Program to inform culturally safe adaptation for Perth, as part of the broader adaptation process. It shows how First Nations guidance can identify cultural and place‐based considerations that may not be apparent from the source program and how this guidance can inform culturally safe adaptation of an evidence‐informed program for a new First Nations context within a prevailing public healthcare model.

The aims of this study were, first, to identify Aboriginal community representatives' perspectives on potential barriers and enablers to recipient engagement and program implementation, and second, to identify cultural and place‐based considerations to inform the culturally safe adaptation of Whānau Pakari for delivery as the Healthy Lifestyle Program in Perth, WA.

## 2 Methods

### 2.1 Study Design

A qualitative study design was used within a broader participatory action research (PAR) approach, informed by co‐design and consumer and community involvement [31, 37]. The PAR approach emphasised collaboration between community members with lived experience and researchers, and shared decision‐making to generate knowledge and inform practice [43]. Data were collected through a single large workshop comprising semi‐structured whole‐group discussions and five facilitated breakout groups to ensure all voices were captured. Consistent with co‐design as the meaningful involvement of end users [44], Aboriginal advisors guided program adaptation in the Perth context during the workshop. The workshop format minimised the burden on participants and enabled gathering a broad range of views in one event, whilst also collating practicable actions and considerations within a short timeframe. It also facilitated a connection between the participants and the program research and clinical team, prioritising the voice of the participants in the process. PAR was sustained through the ongoing involvement of a Cultural Advisory Group.

### 2.2 Participants

Participants were Aboriginal advisors interested in guiding the cultural safety and local adaptation of a healthy lifestyle program for children and young people, with direct or indirect experience of trying to achieve healthy lifestyle change, and an interest in the health and wellbeing of Aboriginal and Torres Strait Islander families and communities. EMHS had an established Aboriginal Health Community Advisory Group (AHCAG) at the time, which met at a tertiary hospital site within the EMHS catchment and comprised Aboriginal community members who provided cultural governance and community oversight for health service initiatives aimed at improving Aboriginal health and wellbeing [45]. The group advised the health service on community priorities and key issues affecting local Aboriginal people, and members fed back information from meetings to their broader community networks [45]. Y.A. attended and presented at a meeting with the AHCAG to provide an overview of Whānau Pakari and describe the proposed program adaptation. This engagement with the AHCAG confirmed that healthy lifestyle education was a priority for the local Aboriginal community and that a model similar to Whānau Pakari had the potential to provide the necessary and appropriate care. The AHCAG also supported collective discussion in a workshop as the method of data collection. At this meeting, group members were invited to participate in the workshop and provided with the AHCAG contact person at EMHS to register their interest. Further information was provided to prospective participants by the Aboriginal Health team at EMHS (one non‐Aboriginal contact with Aboriginal lead oversight). Purposive sampling aimed to recruit Aboriginal community members with interest or experience relevant to healthy lifestyle programs and/or Aboriginal health in the EMHS catchment. Snowball sampling was conducted and welcomed from initial contacts, with group members being invited to share the workshop information and facilitator contact details through their networks. The workshop was scheduled at the same venue as regular AHCAG meetings to minimise participant burden. Participants provided informed consent on the day of the workshop and were reimbursed for their time and travel costs based on Health Consumers' Council WA guidelines. Participants were identified as Elders, where they were recognised within their community as respected leaders and custodians of traditional knowledge. The term ‘recipients’ will henceforth be used to refer to children and young people enrolled in the program, as opposed to the term ‘participants’, which will be used to refer to workshop participants.

### 2.3 Data Collection

The workshop was conducted in April 2024, prior to program inception. A Health Consumers' Council WA Aboriginal Engagement Lead (T.H.) facilitated the workshop. Three members of the research and clinical team (S.P., J.B. and Y.A.) supported the facilitator, alongside members from the program clinical team and the Aboriginal Health team at EMHS. The workshop started with the background of Whānau Pakari and covered the rationale for the adaptation of Whānau Pakari to Perth and the aims of the workshop. The workshop lasted approximately 3 h and consisted of whole‐group discussions followed by five breakout table discussions with a co‐facilitator on each table, with the Aboriginal Engagement Lead facilitator overseeing all discussions. Data collection included digital audio recordings using dictaphones, notes taken on a whiteboard by a co‐facilitator and notes taken on butchers' paper by breakout groups. See Supplementary Table S1, Additional File 1 for the workshop schedule and questions asked.

### 2.4 Expert Involvement and Cultural Governance

Expert involvement in this study included partnership with Health Consumers' Council WA, Aboriginal investigators in the broader Healthy Lifestyle Program research team and cultural governance through an Aboriginal Cultural Advisory Group. Health Consumers' Council WA, an independent, not‐for‐profit organisation that aims to ensure that consumers are at the centre of WA's healthcare system, provided expertise in consumer and community involvement and Aboriginal engagement, including partnership for workshop design and leading facilitation.

Following the workshop, a Cultural Advisory Group was formed to provide ongoing Aboriginal community oversight for the broader Healthy Lifestyle Program research and implementation program, ensuring cultural integrity and alignment with community priorities. The group consisted of 12 Aboriginal Elders who completed an expression of interest after the workshop to provide cultural guidance to the program. The group's purpose included oversight and advice on cultural safety, community priorities, program adaptation and interpretation of research findings.

### 2.5 Data Analysis

Audio was transcribed verbatim by a professional transcription service, with a confidentiality agreement in place. S.P. reviewed the recording against the transcript and ensured the transcript was accurate and anonymised, with further review by T.H. and J.B. where there was uncertainty regarding what was said. Notes taken on the whiteboard and butchers' paper were also transcribed for analysis. Lumivero's NVivo 14 was used to manage, index, code and map the data [46]. During the whole‐group discussion, contributions occurred in an open forum with multiple participants speaking, and it was not possible to reliably distinguish individual speakers in the audio recording. Quotes from the whole‐group discussion are therefore only attributed by participant sex, which could be reliably determined, rather than an individual participant number. In contrast, as breakout table discussions occurred in smaller facilitated groups, this allowed reliable differentiation between individual participants; quotes from these discussions are attributed using table‐specific participant numbers alongside sex.

To identify potential determinants of recipient engagement and program implementation, data were analysed using a Framework Analysis approach [47], employing the updated Consolidated Framework for Implementation Research (CFIR) as the theoretical framework [48]. The analysis was based on Smith, J. et al.'s barrier and enabler analysis [49], incorporating modifications from Smith, S. et al. [50]. This approach was selected because it provided a structured but flexible method for identifying implementation determinants across multiple contextual domains. The CFIR includes 48 constructs and 19 subconstructs across five domains: the innovation being adapted (defined as the Healthy Lifestyle Program), the outer setting (defined as the Perth and WA community and WA healthcare system), inner setting (defined as CACH), characteristics of individuals, and implementation process considerations [48]. The CFIR was also chosen to support comparability across related engagement and evaluation activities for the Healthy Lifestyle Program. Specifically, determinants identified in this study will be compiled with determinants identified in separate but related studies with key partners and consumers and incorporated into an Implementation Research Logic Model [51]. This enables multiple perspectives to be collated using a shared framework to inform ongoing adaptation and implementation planning.

Transcriptions and annotations were deductively coded to the CFIR to systematically identify determinants relevant to engagement and implementation. Regular meetings were held with S.P., S.S., J.M., T.H. and Y.A. during the analysis process. Through feeding back preliminary results to the Cultural Advisory Group, it became evident that, while the CFIR and its constructs provided analytical structure for identifying and classifying the determinants (and will be used in the future logic model), the framework was not easily relatable/translatable in ways that resonated with the Cultural Advisory Group. Therefore, we further inductively classified the identified CFIR determinants' initial labels into key themes and sub‐themes, relating to cultural and place‐based considerations for program adaptation. This ensured the findings remained grounded in participants' language and experiences to support interpretive depth, enhance participant relatability and prioritise Aboriginal and Torres Strait Islander voice. Identified themes and sub‐themes were reviewed and endorsed by the Cultural Advisory Group. Table 1 outlines the analytical process in more detail. Two members of the Cultural Advisory Group were nominated by the group and reviewed the final draft paper. Processes and reporting were conducted in line with the Standards for Reporting Qualitative Research guidelines [52].

**Table 1:** Analytical process and description using Framework Analysis with the Consolidated Framework for Implementation Research [47, 48, 49, 50].

Framework Analysis stage	Description
1. Familiarisation	S.P. immersed in the data, including listening and re‐listening to the audio recordings, making summary notes, ensuring the transcription was accurate, and reading and re‐reading the transcription.
2. Identifying a thematic framework	All 48 constructs and 19 subconstructs across the five domains of the updated CFIR (Innovation, Outer Setting, Inner Setting, Individuals and Implementation Process domains) were reviewed as part of the thematic framework [48, 49, 50].
3. Indexing	Transcriptions and annotations were deductively coded in NVivo 14 by S.P. to the CFIR domains and constructs, captured as short statements, sentences or short conversations. Iterative revisions were undertaken, resulting in data being recoded to another relevant construct. Regular meetings were held with S.P., S.S., J.M., T.H. and Y.A. to explore themes, coding and potential crossover between constructs.
4. Charting	Charting was based on an adapted version of Smith, S. et al.'s (2023) guidelines for determining barriers and enablers [50]. Data were categorised as either a potential barrier or an enabler within each construct. Theme labels were used to capture the topic of the data capture (statement, sentence(s), etc.).
5. Mapping and interpretation	S.P. developed initial themes based on the determinant labels of each CFIR construct. Themes were further developed inductively and through an iterative process with S.S. and Y.A. and reviewed with the wider research team for participant relatability to support communication and feedback with participants and the community. Figure 1 provides an overview of the reclassification of the CFIR determinants to the eventual themes/sub‐themes. Findings were regularly presented to the Cultural Advisory Group throughout the analysis process. Regular updates on the findings were also presented to the wider Healthy Lifestyle Program Investigator Team.

Abbreviation: CFIR, Consolidated Framework for Implementation Research.

### 2.6 Researcher Characteristics and Reflexivity

As outlined in the protocol for the larger project, ‘the research team aspires to achieve research that is “by‐community‐for‐community.” It involves diverse researchers and advisors from transdisciplinary backgrounds, with a commitment to strength‐based research that prioritises and privileges child, young people, and family voices alongside the community. The group have a commitment to working towards health equity and working in genuine meaningful partnership’ [37, p. 14].

This team comprises two Aboriginal and four non‐Aboriginal researchers with complementary skillsets and experience. The team holds significant transdisciplinary expertise in child health, consumer and community involvement, strengths‐based community healthy lifestyle program delivery, paediatrics, health psychology, health promotion, health systems change and implementation, implementation science, and qualitative and mixed‐methods research. Further details about the roles of the research team are outlined in Supplementary Appendix 1, Additional File 1.

S.P. kept an audit trail throughout the data analysis process via NVivo, and reflexive annotations/journaling were frequently drawn upon, added to and reviewed during the iterative analysis process. Regular reviews and discussions of the analysis took place with S.P., S.S., T.H. and Y.A. providing further interpretation and insight and revision of the themes and sub‐themes. The broader team also met regularly to discuss the findings during development.

## 3 Results

A total of 29 participants were recruited from various Aboriginal community groups (23 female, 79%), with 28 being Elders (97%), to attend the workshop in April 2024.

### 3.1 Determinants

Table 2 outlines the 44 program determinants (16 barriers and 28 enablers), classified within 18 constructs across all 5 domains of the CFIR. Determinants covered both the Whānau Pakari model (innovation characteristics and delivery model) and the local Perth context (outer and inner settings and local individual characteristics), informing program components that may need to change when adapting the program locally.

**Table 2:** Barriers and enablers identified in the workshop, classified within the Consolidated Framework for Implementation Research for the Healthy Lifestyle Program [48, 49, 50].

CFIR domain and construct(s) [48]	Barrier or enabler	Determinant label^a^
1. Innovation: The Healthy Lifestyle Program		
1G. Innovation Design: How the innovation is designed and packaged, including how it is assembled, bundled and presented.	Enabler	1Ga. Inclusive program. 1Gb. Culturally secure program. 1Gc. Clear participation guidelines. 1Gc. Sticking to the schedule. 1Gd. Specific program content recommendations for healthy lifestyle education
2. Outer Setting: Perth/WA Community, WA Healthcare System		
2B. Local Attitudes: Whether sociocultural values and beliefs encourage the Outer Setting to support implementation and/or delivery of the innovation	Barrier	2Ba. Interpersonal and institutionalised racism. 2Bc. Mistrust of the healthcare system or the government
	Enabler	2Ba. Healing and self‐determination. 2Bb. Reclaiming knowledge and culture
2C. Local Conditions: Whether economic, environmental, political and/or technological conditions enable the Outer Setting to support implementation and/or delivery of the innovation.	Barrier	2C. Limited access to health‐promoting environments and traditional ways of living
2D. Partnerships & Connections: Whether the Inner Setting is networked with external entities, including referral networks, academic affiliations and professional organisation networks.	Enabler	2D. Encourage positive community connections for families
3. Inner Setting: CACH		
3A. Structural Characteristics—1. Physical Infrastructure: Whether the layout and configuration of space and other tangible material features support the functional performance of the Inner Setting.	Barrier	3A1a. Inappropriate venue location
	Enabler	3A1b. Comfortable or familiar venue, including at parks or on Country
3A. Structural Characteristics—3. Work Infrastructure: Organisation of tasks and responsibilities within and between individuals and teams, and general staffing levels, supports the functional performance of the Inner Setting.	Barrier	3A3. Limited staff numbers may impact the program if staff are unwell
3D. Culture—1. Human Equality‐Centredness: Whether there are shared values, beliefs and norms about the inherent equal worth and value of all human beings.	Barrier	3D1. Cultural differences between families and staff
	Enabler	3D1. Promote mutual respect for all involved with the program
3D. Culture—2. Recipient‐Centredness: Whether there are shared values, beliefs, and norms around caring, supporting and addressing the needs and welfare of recipients.	Enabler	3D2. Create a safe and trustworthy environment that prioritises the recipient's voice
3D. Culture—3. Deliverer‐Centredness: Whether there are shared values, beliefs and norms around caring, supporting and addressing the needs and welfare of deliverers.	Enabler	3D3. Prioritise safety for staff
3J. Available Resources—1. Funding: Whether funding is available to implement and deliver the innovation.	Barrier	3J1. Funding for programs is often not sustained
3K. Access to Knowledge & Information: Whether guidance and/or training is accessible to implement and deliver the innovation.	Enabler	3Ka. Train staff to communicate with compassion, respect and non‐medical language. 3Kb. Train staff to deliver culturally secure practice
4. Individuals		
4I. Innovation Recipients—A. Need: Whether the individuals who are directly or indirectly receiving the innovation have deficits related to survival, wellbeing or personal fulfilment, which will be addressed by implementation and/or delivery of the innovation.	Enabler	4IA. There is a need for families to be educated about components of a healthy lifestyle
4I. Innovation Recipients—C. Opportunity: Whether individuals who are directly or indirectly receiving the innovation have the availability, scope and power to do so.	Barrier	4IC. Busy lifestyle and timing of the program. 4IC. Difficulties with family structure or conflict within the family. 4IC. Means of transport and the cost of getting to the program. 4IC. Physical health issues reducing access to the program. 4IC. Financial and food insecurity. 4IC. Drug and alcohol use
4I. Innovation Recipients—D. Motivation: Whether individuals who are directly or indirectly receiving the innovation are committed to doing so.	Barrier	4IDa. Cultural differences or conflict between recipients or families. 4IDb. Lack of motivation to engage (either parents or recipients). 4IDb. Intergenerational trauma and pre‐existing mental health conditions
5. Implementation Process		
5A. Teaming: Whether individuals join together, intentionally coordinating and collaborating on interdependent tasks, to implement the innovation.	Enabler	5A. Partner with community organisations. 5A. Form an Elders advisory group to provide ongoing input into the program
5B. Assessing Needs—2. Innovation Recipients: Whether individuals collect information about the priorities, preferences and needs of recipients to guide implementation and delivery of the innovation.	Enabler	5B2. Conduct background cultural research before meeting families or forming groups
5E. Tailoring Strategies: Whether individuals choose and operationalise implementation strategies to address barriers, leverage enablers and fit context.	Enabler	5E. Provide budget support and financial counselling. 5E. Link families with free or reduced‐cost food options. 5E. Increase availability of sessions, including with home visits, rotating venues, adapting to other time commitments, etc. 5E. Provide alternative transport options, either directly or by linking with other organisations. 5E. Mental health support and building self‐esteem. 5E. Help families establish routines and schedules
5F. Engaging—2. Innovation Recipients: Whether individuals attract and encourage recipients to serve on the implementation team and/or participate in the innovation.	Enabler	5F2a. Engage with parents and the wider family unit. 5F2b. Session reminders (SMS, email, mail and fridge brochures) and regular contact with families. 5F2b. Make the program appear interesting and engaging and offer incentives. 5F2b. An inspirational mentor to help increase motivation to engage

Abbreviations: CACH, Child and Adolescent Community Health; CFIR, Consolidated Framework for Implementation Research; WA, Western Australia.

^a^ Determinant number and letter codes correspond to Figure 1.

Potential barriers were frequently identified in the Individuals domain, relating to difficulties families may experience with engagement and access, largely reflecting relational, systemic and environmental factors. Barriers also sat in the Outer Setting, relating to the cultural context of families within the program, including racism, mistrust of the healthcare system and limited access to health‐promoting environments, and in the Inner Setting, relating to the venue, funding and workforce.

Potential enablers predominantly related to the Innovation Design and the Implementation Process, including tailoring and engagement strategies such as increasing accessibility of the program, partnerships and communication with families.

### 3.2 Reclassification Into Themes

**Figure 1:**
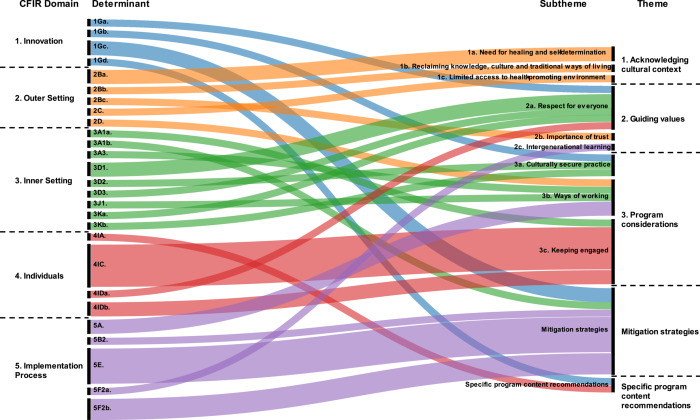
Thematic map of the CFIR domains and determinants to the final themes and sub‐themes. Determinant codes correspond to CFIR constructs and determinants (barriers and enablers) in Table 2. Abbreviation: CFIR, Consolidated Framework for Implementation Research.

Upon reclassification into relatable themes, three themes were identified: *acknowledging cultural context*, *guiding values* and *program considerations*, each with sub‐themes, as outlined in Table 3. Figure 1 visually maps the relationship between the CFIR determinants and the derived themes and sub‐themes.

**Table 3:** Identified cultural and place‐based considerations for adapting the Healthy Lifestyle Program for Perth, Western Australia.

Theme	Sub‐theme	Example quote
Acknowledging cultural context		
	Need for healing and self‐determination	*‘I think the issue that we see over here is that we're trying to have a voice, but there's a lot of, um, hatred and ugliness’.* – Table 1, Participant 4 (Male)
	Reclaiming knowledge, culture and traditional ways of living	*‘Well, I look at it like the culture never left us. We left culture…. And we have to get back there. We have to get back there’.* – Table 1, Participant 4 (Male)
	Limited access to health‐promoting environments	*‘this is the problem that we have in [suburb 1] or other places like [suburb 2] or even [suburb 3]. They're the poor areas, and they got one shopping centre there. They've got all the takeaways, all that greasy food. Not a lot of health options. We got to go miles to do shopping’.* – Table 5, Participant 5 (Female)
Guiding values		
	Respect for everyone	*‘At the end of the day, we're different. We got our own way. They [non‐Aboriginal people] got their own way…. A little bit of kindness and respect goes a long way’.* – Table 4, Participant 2 (Female)
	Importance of trust	*‘Maybe they don't trust the system. Maybe they don't trust the system like they don't trust the school system because of what happened to the Stolen Generation* a *. They don't trust the government because they govern whatever, for whatever reason. The moment of trust, people coming into their home, like, “Who are you? Why are you coming into my house for?… You're the government. What are you going to do? Are you doing the right thing by us? Are you not doing the right thing by us?”’* – Table 2, Participant 1 (Female)
	Intergenerational learning	*‘I would be utilising parents, grandparents, uncles and aunties. Let's bring them on board, share their knowledge, how they work with their kids and their grandkids. And I know a lot of that happens around. So, we need to start utilising some of those people, sharing their skills and their knowledge’.* – Whole‐group discussion (Female)
Program considerations		
	Culturally secure practice	*‘Cultural training…[the program staff] have to learn about the fact that, we pick up straight away…. The body language stuff. We pick up on that straight away’.* – Table 4, Participant 2 (Female)
	Ways of working	*‘Utilise existing resources, see what they've got, what they do, how can we help them or provide assistance for them to be able to do what they need to do?’* – Table 2, Participant 3 (Female)
	Keeping engaged	*‘The venue. A lot of our mob, there's certain venues they don't like going to’.* – Table 4, Participant 2 (Female)

^a^ ‘Stolen Generation’ refers to a time in Australian history when Aboriginal children were forcibly removed from their families due to government policies.

#### 3.2.1 Acknowledging Cultural Context

We[‘re] struggling against a colony that come to conquer our world. We try to come out of it on top and they've held us here.

Table 1, Participant 5 (Male)

This theme outlines the importance of awareness and acknowledgement of the cultural context of recipients of a healthy lifestyle program. This theme highlights the need for program adaptation to account for racism and intergenerational trauma, recognise the importance of culture and traditions, and consider the reality of environments that can contribute to difficulties with healthy living for Aboriginal and Torres Strait Islander people.

##### 3.2.1.1 Need for Healing and Self‐Determination

Participants detailed specific personal examples of experienced interpersonal racism, as well as broader examples of struggles with institutionalised racism and the need for healing of intergenerational trauma. This evolved into discussions around the importance of self‐determination in the healing process.

I think the issue that we see over here [in Australia] is that we're trying to have a voice, but there's a lot of, um, hatred and ugliness.

Table 1, Participant 4 (Male)

In a broader discussion about the ongoing effects of colonisation and institutional barriers, participants described the need to receive additional support to overcome systemic inequities and work towards healing and self‐determination.

And we need all the support we can to find our feet. Just like everyone else, but it's a bit hard when you constantly get put down or pushback beside it all the time.

Table 1, Participant 4 (Male)

Participants articulated a broader need for safe spaces for Aboriginal and Torres Strait Islander people, including within the healthcare system. Although not raised as a specific Healthy Lifestyle Program adaptation, this highlighted the importance of delivering care in settings experienced as safe, trusted and culturally appropriate.

We need our own Aboriginal Noongar^1^ Centre…. We need our own. We don't want to hire halls or venues. They've gotta start…the government…they've gotta build Noongar Centres in all the different suburbs’.

Table 1, Participant 3 (Male)

##### 3.2.1.2 Reclaiming Knowledge, Culture and Traditional Ways of Living

The importance of reclaiming traditional knowledge and culture and ways of living that had been stripped due to colonisation was discussed. For program adaptation, this highlighted that healthy lifestyle education should not only provide nutritional and physical activity advice, but also create opportunities, where appropriate, to recognise Aboriginal cultural knowledge, family knowledge‐sharing and traditional ways of living.

Well, I look at it like the culture never left us. We left culture…. And we have to get back there. We have to get back there.

Table 1, Participant 4 (Male)

Participants identified that traditional knowledge‐sharing had been jeopardised, with more pressure being placed on Elders and grandparents to share this knowledge, with younger generations lacking the same opportunities for receiving knowledge. Participants also emphasised the importance of supporting Elders and grandparents in reclaiming knowledge sharing to younger generations.

The biggest problem there is we must get the parents on board. We as grandparents, we're not going to be around forever, and too much pressurising has been put back on our grandparents from all walks of life.

Table 4, Participant 3 (Female)

Yeah, so the younger generation needs to come on board.

Table 4, Participant 5 (Female)

However, it was recognised that Elders were best placed to play a pivotal role in reclaiming and sharing knowledge with younger Aboriginal and Torres Strait Islander people.

So, realistically, we are starting a culture that has been on the ground zero for so long, and it's up to us Elders now to bring our culture up.

Table 1, Participant 4 (Male)

It was noted that the local Aboriginal language and dialect were not being spoken as commonly and that it was important for Aboriginal and Torres Strait Islander recipients and their families to be supported in reclaiming the culture that had been taken through epistemicide.

Identifying your language, identifying your culture, identifying your totem^2^, and identifying your mob^3^.

Table 1, Participant 4 (Male)

Other identified traditional ways of living that had been silenced included speaking the local Aboriginal language and dialect; lighting fires^4^; and the use of traditional foods, bush tucker^5^ and bush medicines^6^. These examples of traditional ways of living were relevant to program adaptation because they indicated the potential importance of incorporating culturally meaningful understandings of food, health and connection to culture into healthy lifestyle education where relevant.

Because back in the day, no matter what, before white man come, every culture, Indigenous, had their own diet…they lived by that over the years.

Table 1, Participant 5 (Male)

##### 3.2.1.3 Limited Access to Health‐Promoting Environments

Participants also discussed how the stripping of traditional knowledge and culture due to colonisation had, in part, resulted in limited access to a health‐promoting environment, thereby increasing the difficulties with Aboriginal and Torres Strait Islander people achieving a healthy lifestyle. Examples included increased screen use, reduced natural physical activity, ease of access to vaping and reduced access to clean water. While one participant noted that some schools are making healthier changes with foods served at canteens, the consensus from participants was that low‐nutrition foods were still far more affordable and accessible than healthier and traditional options.

It's having access to food. If you don't have access to those foods, you can't buy them.

Table 2, Participant 3 (Female)

this is the problem that we have in [suburb 1] or other places like [suburb 2] or even [suburb 3]. They're the poor areas, and they got one shopping centre there. They've got all the takeaways, all that greasy food. Not a lot of health options. We got to go miles to do shopping.

Table 5, Participant 5 (Female)

These difficulties were seen to be compounded by a reduction in knowledge and health literacy of some Aboriginal and Torres Strait Islander families, highlighting the need for education in this area.

What about their educational background? They might not be able to understand what's going on.

Table 2, Participant 1 (Female)

Low literacy or cognitive understanding of nutritional labels.

Table 2, Butchers' paper notes

Whilst the notion of food sovereignty was not directly discussed, the need to ensure access to and knowledge of traditional food sources was emphasised.

When adapting Whānau Pakari for Perth, these findings highlight the need for locally relevant trauma‐aware, culturally safe approaches that account for racism and mistrust and for program delivery settings that feel safe, welcoming and culturally appropriate. These findings also highlight practical considerations relating to barriers to healthy living that need to be considered when adapting the program curriculum.

### 3.3 Guiding Values

At the end of the day, we're different. We got our own way. They [non‐Aboriginal people] got their own way…. A little bit of kindness and respect goes a long way.

Table 4, Participant 2 (Female)

This theme outlines key guiding values that were identified as critical components of a healthy lifestyle program adaptation for Perth. These values included mutual respect, the importance of staff earning trust from recipients, and intergenerational learning. For the adapted program, participants' emphasis on these values highlights the importance of staff communication approaches, building relationships over time and delivery formats that include wider family networks where appropriate. These findings also suggest that facilitation of group settings may involve managing potential interpersonal or inter‐family tensions while still maintaining inclusivity.

#### 3.3.1 Respect for Everyone

Cultivating an environment of respect was deemed crucial for program adaptation. As the program aimed to prioritise Aboriginal and Torres Strait Islander families, it was identified that conflict between recipients and families could contribute to reduced recipient retention within the program. This was seen as potentially, in part, due to ‘family feuding’ between different Aboriginal and Torres Strait Islander family groups.

Just look at their safety. And you need to identify your family groups and be aware.

Table 3, Participant 4 (Female)

Be aware of the differences in family groups because every nation has it. Awareness.

Table 3, Participant 1 (Female)

And be aware of the cultural differences, because some groups have things in common and some of them don't. Be aware of the cultural differences. Well, we should always find out.

Table 3, Participant 1 (Female)

The potential for broader conflict was also considered, due to having families from different nationalities or ethnic backgrounds together for group sessions.

If you put a whole lot of, um, people together from different nations or whatever, there's going to be cultural language barriers, uh, cultural um understandings, misunderstandings.

Table 1, Participant 4 (Male)

When asked whether this meant that group sessions should run with Aboriginal and Torres Strait Islander families separately, participants replied that group sessions should be inclusive, for people of all backgrounds.

Because we're multi…, you know, we're multicultural here in Australia, you know? So we've got to do it that way.

Table 1, Participant 5 (Male)

Yeah, that thing, so you can give each culture that sense of pride, of knowing that they're in control of a future or a destination or project that allows them to want to engage with other people.

Table 1, Participant 4 (Male)

An environment of mutual respect was seen to be a key factor in overcoming potential conflict between families, as well as between families and program staff. Key to this was the importance of training staff to communicate with compassion, respect and non‐medical language. Conversely, participants also highlighted the importance of ensuring program recipients and families demonstrate respect to program staff.

Two‐way respect because you have to show respect to staff, regardless of who or what nationality or whatever, etcetera.

Table 4, Participant 3 (Female)

#### 3.3.2 Importance of Trust

The importance of trust was also identified as critical to program success. Difficulties in this area included the past experiences of systemic and institutionalised racism, which may contribute to Aboriginal and Torres Strait Islander families having difficulty trusting program staff. This was not the only reason identified for potential mistrust to develop:

Maybe they don't trust the system. Maybe they don't trust the system like they don't trust the school system because of what happened to the Stolen Generation^7^. They don't trust the government because they govern whatever, for whatever reason. The moment of trust, people coming into their home, like, “Who are you? Why are you coming into my house for?… You're the government. What are you going to do? Are you doing the right thing by us? Are you not doing the right thing by us?”

Table 2, Participant 1 (Female)

This highlighted the importance of program staff being aware of the potential for mistrust and persisting to work to earn trust from families.

#### 3.3.3 Intergenerational Learning

Intergenerational learning was also highlighted as a guiding value, particularly for Aboriginal and Torres Strait Islander families. Despite the previously mentioned difficulties Elders and grandparents described with pressure being placed on them due to the loss of knowledge sharing, multiple groups identified the importance of including the wider family unit within the program, due to the influence that the wider family has on individuals within Aboriginal and Torres Strait Islander families.

I would be utilising parents, grandparents, uncles, and aunties. Let's bring them on board, share their knowledge, how they work with their kids and their grandkids. And I know a lot of that happens around. So, we need to start utilising some of those people, sharing their skills and their knowledge.

Whole‐group discussion (Female)

We need to use the parents, the grandparents to teach them, pass down their skills and knowledge.

Table 2, Participant 3 (Female)

### 3.4 Program Considerations

so every client needs to be valued and needs to be felt like there is a place for them to be seen.

Table 1, Participant 4 (Male)

This theme covers the multiple cultural and place‐based considerations for successful program functioning and operation. Outlined are culturally secure practices and creating a safe and welcoming environment, key ways of working, and the need for program staff to keep engaged with families.

#### 3.4.1 Culturally Secure Practice

Participants emphasised the importance of culturally secure practice within the healthy lifestyle program, with a key aspect of this being the need for training culturally secure staff. Multiple groups identified the necessity for staff to complete cultural awareness training and acknowledge the traditional owners of the land where the program was to be conducted.

Cultural training…[the program staff] have to learn about the fact that, we pick up straight away…. The body language stuff. We pick up on that straight away.

Table 4, Participant 2 (Female)

You've got to acknowledge whose Country you're on from the start before you do anything else.

Table 3, Participant 5 (Female)

However, recommendations went beyond this to include staff identifying their own culture and roots, adopting an awareness of the local culture and history, embracing the local Noongar language, communicating respectfully in a culturally appropriate manner, and wearing clothing with Aboriginal design.

One of the things I found was the communication and understanding our interpretation of the language is different to you, your interpretation.

Table 3, Participant 4 (Female)

This is for a non‐Indigenous understanding of culture, language, and diversity within…. And all in that they've got their own, um, traditional groups inside of that…. So that's the diversity that comes into Aboriginal culture.

Table 5, Participant 5 (Female)

Other aspects of culturally secure practice included creating a safe and trustworthy environment that prioritises recipient voice, organising cultural camps with Elders, running cooking sessions on traditional food with Aboriginal or Torres Strait Islander cooks, and conducting healing circles.

We can keep our dampers^8^, we can keep our kangaroo stew, we can keep anything we want as long as it's in the budget, and it's healthy for us.

Table 1, Participant 4 (Male)

Kangaroo stew…we can teach the kids to work with the adults to learn how to cook it.

Table 3, Participant 1 (Female)

#### 3.4.2 Ways of Working

Some key considerations for ways of working were also identified. This included partnerships with various community groups, such as engaging with community organisations or groups to assist with program promotion or engagement.

Maybe if, this program looks for sponsors…. And that has to come from this program. They've got to do that.

Table 4, Participant 3 (Female)

More community networking (lever off past/existing programs)

Whole group, whiteboard notes

Community partnership also included working to encourage positive community connections for recipients and their families: between individual families as well as between families and community organisations and initiatives. Participants also recommended forming a Cultural Advisory Group of local Elders to continue to advise the program.

Are you starting an advisor group for this whole program? Are you starting a community engagement like what's here [at the location of the workshop]?

Table 1, Participant 5 (Male)

Participants emphasised the importance of ensuring the program was adequately resourced, including working with previously mentioned community partners to make use of pre‐existing resources.

Utilise existing resources, see what they've got, what they do, how can we help them or provide assistance for them to be able to do what they need to do?

Table 2, Participant 3 (Female)

Are yous gonna organise it in a way where the person who's running the program on the day, if something happens to them, they get sick…?

Table 4, Participant 2 (Female)

Participants stressed the need to work hard to secure long‐term funding, so the program could be embedded into the health system should the pilot demonstrate success. Reflecting the need for program staff to acknowledge the context of Aboriginal and Torres Strait Islander families and their need for healing and self‐determination, participants expressed frustration with their experience of programs designed with and for Aboriginal families rarely proceeding beyond the pilot stage.

Funding. Everything that started up around here, the funding…is cut. No matter how successful the program is, around fifty percent the funding is cut.

Table 4, Participant 2 (Female)

#### 3.4.3 Keeping Engaged

Another program consideration identified related to keeping engaged with recipients and families in the program. Multiple barriers to recipient engagement and retention were identified, including busy lifestyle, financial and food insecurity, difficulties with family structure, drug and alcohol use, competing priorities, reduced motivation, intergenerational trauma and pre‐existing mental health conditions, and accessibility and location of sessions.

The venue. A lot of our mob, there's certain venues they don't like going to.

Table 4, Participant 2 (Female)

Example quotes regarding these potential barriers to engagement are included in Supplementary Table S2, Additional File 1. Participants also identified mitigation strategies to these barriers, including conducting background cultural research; comfortable and familiar venues; increasing accessibility of sessions; supporting families with food, transport, budgeting and routine; session reminders; ground rules and sticking to schedule; mental health support; and strategies to increase motivation to attend. Example quotes relating to these strategies, and the resulting adaptations made to the Healthy Lifestyle Program, are included in Supplementary Table S3, Additional File 1, which directly respond to many of the barriers in the CFIR domains, especially those in the Individuals and Inner Setting domains.

Many recommendations were made for content to include in the program curriculum, as outlined in Table 4.

**Table 4:** Recommended content for the Healthy Lifestyle Program curriculum.

Recommended program content	Example quote
Healthy meal plan, shopping, label reading and cooking	*‘To work with a food, um, nutritionist who sets, to have a set meal for dinner, lunch, or breakfast. Go with the nutritionist to the shop and buy all the items and then cook the dinner’.* – Table 1, Participant 4 (Male) *‘Can you take kids into the shop and get them to read labels?’* – Table 2, Participant 2 (Female)
Growing food through school and community gardens	*‘Um, encouraging the schools and the community to do their own gardens. That's not hard to do. But how to look after the garden. How to maintain it. Even, even growing your own bush tucker* a *. And there's quite a few places that are doing that now. And, um, so we need to start looking at that. And some of us are, you know, in our own backyard, we're doing it [creating a garden]. But we need to encourage young ones’.* – Whole‐group discussion (Female)
Increasing physical activity time and reducing screen time	*‘So um, maybe, more. Yeah. Less technology, and more exercise’.* – Whole‐group discussion (Female)
Drug and alcohol education	*‘I thought that drugs would be the main topic of what we see in a healthy lifestyle, the drugs and the young people’.* – Whole‐group discussion (Female)
Holistic and individualised care	*‘The other one is, ask the kids. Ask the family what they'd like to see happen instead of us dictating all the time to them’.* – Table 3, Participant 1 (Female)

^a^ ‘Bush tucker’ refers to any food native to Australia, traditionally sourced and eaten by Aboriginal and Torres Strait Islander people.

These program considerations highlight program design and delivery priorities for the adapted Perth program, including development of culturally secure staff practice, local governance and partnerships, improved accessibility of the program, and strategies to support retention. Participants also highlighted that sustainable resourcing and long‐term funding are important for community trust and program continuity.

## 4 Discussion

This study presents the perspectives of the Aboriginal community on how an evidence‐informed program, Whānau Pakari, should be adapted to be acceptable and culturally safe for families in Perth. Analysis of workshop data identified 44 potential determinants of engagement and implementation, mapped to the CFIR, which were subsequently reclassified into three participant‐relatable themes: *acknowledging cultural context*, *guiding values* for the program, and specific *program considerations*, so that Aboriginal community priorities could be communicated in plain language while remaining actionable for adaptation and planning. Together, the determinants and themes emphasise that successful adaptation and local implementation of Whānau Pakari is dependent not only on program content, but also on culturally secure relationships and practice, trusted setting(s), and practical approaches that reduce barriers to participation while embedding Aboriginal governance and ongoing partnership. This study highlights the value of identifying Aboriginal community perspectives before implementation when adapting an evidence‐informed program for a new context. Although Whānau Pakari was a plausible model for scale‐out, these findings demonstrate that adaptation requires more than transferring an intervention model but also requires cultural considerations and attention to local history, community priorities and cultural safety.

Several community priorities spoke directly to delivery mechanisms that underpin Whānau Pakari, including optimising accessibility by holding the program in the local community and offering home visits, encouraging a whole‐of‐family approach, holistic care and the overarching healthy lifestyle education curriculum [33]. Other aspects of the program were flagged as requiring modification in order to be appropriate for the Perth community, including considerations around the venue for program delivery, building of trust and cultural safety, how families are brought together through a trauma‐aware welcoming environment, and how specific engagement barriers could be mitigated.

This study showed that past experiences of institutionalised racism were identified as being a potential barrier for Aboriginal and Torres Strait Islander families engaging with this program and trusting healthcare staff. This has been demonstrated in other research relating to healthy lifestyle programs to address childhood obesity in a NZ context [38, 53]. Extensive consultation conducted as part of the First Nations Consultations for the Australian Human Rights Commission also found that many Aboriginal and Torres Strait Islander people experience a range of racist behaviours, including from healthcare providers, and that such behaviours lead to ongoing distrust of the healthcare system and reduce willingness to access health services [54].

This study showed that stripping of traditional knowledge due to colonisation included reduced use of traditional foods and bush tucker, contributing to limited access to a health‐promoting environment, with low‐nutrition foods being more accessible than traditional options. These findings highlight that access to traditional foods may be constrained not only by loss of knowledge but also by loss of practical access, availability and opportunities for intergenerational learning. These findings also extend beyond Aboriginal and Torres Strait Islander families, highlighting the need to address the limited access to health‐promoting environments within which programs operate, as well as broader determinants of health and wellbeing for all families. The broader environment's impact on families must be considered when understanding their ability to adopt persistent healthy lifestyle changes, and nutritional education should include locally relevant strategies, such as linking with community food initiatives and recognising traditional foods if guided by local Aboriginal community members. Participants' emphasis on intergenerational learning also has implications for the delivery of healthy lifestyle program content. Programs may need to allow for storytelling and shared family learning for cultural knowledge about food, health and wellbeing to be passed on, rather than solely providing education through health professionals. As highlighted by participants, this should be governed locally, with care not to place an unpaid responsibility on Elders or grandparents who are already feeling significant pressure in this area.

Like this study, the First Nations Consultations for the Australian Human Rights Commission found that many Aboriginal and Torres Strait Islander people place importance on the need for mandatory cultural safety education for healthcare workers, including training on First Nations health perspectives [54]. Coffin's model of cultural security highlights not only the importance of cultural awareness and safety training, but also the need to progress to culturally secure interactions in order to achieve greater positive impact for Aboriginal and Torres Strait Islander families [55]. Similar to NZ research, participants identified culturally secure, compassionate and respectful care as a potential enabler to support engagement and mitigate the impact of racism on engagement [38].

Constrained resources and funding limitations have been identified as likely barriers to program success for other healthy lifestyle programs [56], and predicted barriers to recipient engagement, including competing priorities and financial and food insecurity, have also been identified in other literature [53]. Participants in this study also identified that some families may perceive addressing their child's pre‐existing mental health conditions or drug and alcohol use to be a higher priority than addressing weight‐related issues and adopting a healthy lifestyle, consistent with previous research elsewhere [53].

The CFIR was originally developed for use with personnel implementing or delivering an innovation and is not often used for collecting data from recipients or the community [57]. However, we have used the CFIR here to structure the barriers and enablers identified by the community because Aboriginal community guidance was intended to inform implementation decisions within the wider research program. Reclassifying CFIR determinants into relatable themes helped keep the findings actionable for implementation while preserving participant voice.

Strengths of this study include the strong community and Elder representation, with a diverse range of experience, the broad range of topics covered and many feasible recommendations being identified. The use of a single large workshop allowed for whole‐group discussions where participants could build on each other's ideas, with subsequent facilitated breakout groups to promote equitable participation and support inclusion of quieter voices and deeper discussion of topics. This was an efficient format to gather diverse perspectives. Another strength is the unique methodological approach, balancing implementation planning with community relatability. Using the CFIR in Framework Analysis identified barriers and enablers in a structured way [47, 49, 50], while presenting the findings as relatable themes, supported communication and community feedback. This approach may be useful in other pre‐implementation engagement processes elsewhere in Australia and internationally, where researchers need to produce findings that are both helpful in implementation and understandable by the communities who contributed them.

Study limitations include some data loss due to technical challenges with audio quality, difficulty attributing comments to individuals in the whole‐group discussions, and multiple group discussions occurring concurrently in a community venue. With the strong Elder representation, the perspectives of children and young people and their parents are under‐represented; however, further research in this area has been undertaken to broaden the perspectives feeding into program adaptation. The facilitated breakout discussions at the workshop were used to mitigate the potential for large‐group formats to amplify power dynamics and to support participation from more withdrawn attendees; however, some perspectives may have still been under‐represented. The discussion also sometimes extended beyond specific Healthy Lifestyle Program adaptations to broader historical and cultural issues affecting Aboriginal communities, which reduced the time available for program‐specific detail. However, these discussions revealed the context in which the program would be implemented and highlighted considerations required regarding cultural safety and trust. Future pre‐implementation engagement should allow space for broader contextual discussion while also enabling facilitators to clearly communicate the scope of the program. While there was some diversity in participant views and disagreement on some issues discussed, this study population was likely representative of the broader local Aboriginal community. Caution is noted, however, in terms of generalisability of results for scaling out or adapting other healthcare services in varying locations, noting the heterogeneity of language groups within Perth and Australia more broadly.

The workshop findings suggest that adapting evidence‐based healthy lifestyle interventions for First Nations communities requires considerations beyond curriculum content. Health services should consider using pre‐existing local cultural governance bodies, where available, for guidance on adaptation and prioritise program delivery in trusted, culturally safe settings with practical supports that reduce access barriers. Healthcare staff capability building should extend beyond one‐off cultural awareness training to more comprehensive development of culturally secure practice, including strengths‐based communication, reflection on power dynamics and systemic racism, and consistent relationship‐building to earn trust over time. The importance of trauma‐aware care in the delivery of such programs is key. Participants' emphasis on program continuity also highlights the importance of sustained resourcing and longer‐term funding as a signal of respect and commitment to the community, particularly given frustration with short‐lived pilot programs. Finally, because many barriers to engagement were framed as structural and environmental, effective adaptation and implementation of programs is likely to require partnership with community organisations and links to broader supports, rather than relying on family education alone. While these findings were developed from local Aboriginal community perspectives, the combination of community involvement with a structured determinant framework and development of relatable themes is a method that can support other under‐represented communities, provided cultural governance is locally led and supported. These findings affirm that culturally safe paediatric healthy lifestyle programs cannot rely on clinical education alone. Policy and healthcare service planning should include practical access supports and sustained program funding as core components of implementation. All services should prioritise those over‐represented in obesity statistics, yet to reach First Nations children, young people and their families, First Nations governance and culturally secure workforce development must be prioritised. For prevention and early intervention, adapting evidence‐informed models should include community involvement in the pre‐implementation process, rather than a ‘lift and shift’ of an ‘off the shelf’ program without place‐based and cultural considerations for the community the program intends to serve.

Further research is being conducted in response to the need to convey the findings of this study to the participants, intended to result in a user‐friendly output. In addition, further planned research includes the development of an Implementation Research Logic Model alongside data from program recipients, health organisation leaders and healthcare professionals [51].

## 5 Conclusions

*Acknowledging cultural context*, *guiding values* and *program considerations* are key to successful implementation of new programs. Collectively, these findings highlight that successful and culturally safe adaptation depends not only on program content but also on the development of culturally secure relationships and practice, trusted and accessible program delivery settings, and practical strategies that support engagement and retention. For health services adapting evidence‐based interventions with First Nations communities, utilising pre‐existing local cultural governance groups from the outset, as well as highlighting the importance of community engagement and partnership prior to (and during) implementation, is key. The findings support investment in comprehensive culturally secure practice training for staff, designing program delivery to optimise access, and planning for program continuity to avoid undermining community trust. Using a structured determinant framework alongside community‐relatable themes may assist other settings to enhance First Nations guidance into translatable implementation actions for program adaptation. Drawing on the wisdom and knowledge of First Nations communities ensures such services are acceptable to the community and culturally safe for recipients, thereby increasing the likelihood that such services will reach those who need them.

## Author Contributions

**Stephen Paull:** project administration, methodology, investigation, data curation, formal analysis, writing – original draft. **Stephanie Smith:** conceptualisation, methodology, formal analysis, validation, supervision, writing – review and editing. **Jordan Bill:** investigation, formal analysis, validation, writing – review and editing. **Joanna C. Moullin:** formal analysis, writing – review and editing. **Tania Harris:** investigation, formal analysis, validation, writing – review and editing. **Yvonne C. Anderson:** conceptualisation, methodology, investigation, validation, supervision, writing – review and editing.

## Disclosure

The funders had no other role in any aspect of the study, including the study design; collection, analysis and interpretation of data; and writing of the manuscript; and in the decision to submit the paper for publication.

## Ethics Statement

Ethical approval has been obtained from the Child and Adolescent Health Service Human Research Ethics Committee (RGS0000006244), the Western Australian Aboriginal Health Ethics Committee (HREC1292), and reciprocal approval from Curtin University Human Research Committee (HREC2024‐0066). Research Governance approval was obtained from the Child and Adolescent Health Service Research Governance (RGS0000006244) and The Kids Research Institute Australia (P354). All methods were performed in accordance with the relevant guidelines and regulations.

## Consent

Written informed consent was obtained through a Participant Information and Consent Form (PICF) for anonymised patient information to be published in this article. For participants who had difficulty with reading, the PICF was read aloud to them, and the consent form was signed in the presence of an independent witness.

## Conflicts of Interest

The authors declare no conflicts of interest.

## Supporting information


Supporting File

## Data Availability

The data that support the findings of this study are not publicly available due to privacy and ethical reasons. The collected data are of a sensitive and personal nature and were collected from participants on the basis that strict confidentiality would be maintained. Data can be available from the corresponding author (S.P.) on reasonable request and will require completion of relevant confidentiality agreements.

## Endnotes

1 ‘Noongar’ refers to the Aboriginal traditional owners of the south‐west region of Western Australia, including Boorloo/Perth, where this study took place.

2 ‘Totem’ refers to a natural object, plant or animal that is a spiritual emblem inherited by Aboriginal and Torres Strait Islander people as part of Aboriginal kinship.

3 ‘Mob’ refers to a group of Aboriginal people associated with a particular family, place or language group.

4 For many Aboriginal cultures, fire has spiritual significance and represents connection to land.

5 ‘Bush tucker’ refers to any food native to Australia, traditionally sourced and eaten by Aboriginal and Torres Strait Islander people.

6 ‘Bush medicine’ refers to traditional medicines derived from natural Australian flora and fauna, used by Aboriginal and Torres Strait Islander people for their healing properties.

7 ‘Stolen Generation’ refers to a time in Australian history when Aboriginal children were forcibly removed from their families due to government policies.

8 ‘Damper’ refers to bread traditionally baked in hot coals. While Aboriginal people have historically made damper from ground seeds, today, damper is typically made with wheat flour.
