# Supplementary material for: Being Heard, Being Valued, Being Understood—Aboriginal Community Perspectives on Adapting a Healthy Lifestyle Program for Boorloo/Perth, Western Australia: A Qualitative Study
Source: Health Expect. 2026 Jul 16;29(4):e70754. doi: 10.1111/hex.70754 (PMC13373932; doi:10.1111/hex.70754)
Supplement: Supplementary file 1 — Supporting File [file HEX-29-e70754-s001.docx]

Supplementary Table 1: Questions and content covered in the workshop

| **Workshop Component** | **Description** |
| --- | --- |
| Opening | Introductions, signing of consent. Background of Whānau Pakari (NZ-based healthy lifestyle program), rationale for adaptation to Perth, and aims of the workshop reviewed |
| Question 1: What would you want to see from a Healthy Lifestyle Program for Aboriginal children and families in Perth? | Whole group discussion  Notes recorded on a whiteboard by co-facilitator and participants |
| Question 2: What are some of the things that might get in the way of families coming to and staying with the program? | Breakout group discussions on five tables  Notes recorded on butchers’ paper  Aboriginal Engagement Lead facilitator reviewed butchers’ paper responses during a break and identified six broad preliminary barriers to recipient engagement |
| Question 3: What are the things that will make it easier for families to attend and keep coming back? | Breakout group discussions  Notes recorded on butchers’ paper on each of the preliminary barriers identified, with subsequent groups only recording new ideas or expanding on pre-existing ones |
| Question 4: What do we need to do to make sure the program is culturally safe for families and that they feel comfortable and welcome? | Breakout group discussions  Notes recorded on butchers’ paper |
| Closing | Participation payment process  Evaluation forms  Expression of interest for the Cultural Advisory Group  Thank-you |

Supplementary Appendix 1: Researcher Characteristics

SP is a medical registrar at Child and Adolescent Community Health specialising in general paediatrics and is undertaking a PhD with this project. He undertook internal and external training in qualitative research and NVivo.

SS is a Senior Research Fellow and Chartered Health Psychologist. Her expertise encompasses qualitative research, health psychology, implementation science, and consumer and community involvement. She oversaw the qualitative methods and process, provided training and guidance on the analysis method, advised on the CFIR domains and constructs and the analysis for this study.

JB is a Yamatji man from Wajarri Country in Western Australia’s Gascoyne region and is a qualified personal trainer as well as an Aboriginal and Torres Strait Islander Primary Health Care Practitioner. He has previously worked with East Metropolitan Health Service as an Aboriginal Health Liaison Officer and Senior Aboriginal Health Promotion Officer. JB knew some of the workshop participants through these previous roles.

JM is an Associate Professor with expertise focused on implementation science and knowledge translation. She advised on the CFIR domains and constructs.

TH is a Bundjalung woman and is the Engagement Manager, Aboriginal Engagement Lead at Health Consumers’ Council, with a long track record of working with consumers and facilitating consumer and community events and workshops. She knew some of the workshop participants through previous and current roles.

YA is a Professor in Paediatrics and Child Health, has a track record of partnering across sectors to deliver innovative solutions to complex health problems and is being supported to champion this work due to her on-the-ground implementation research expertise. As a paediatrician, she has experience working with marginalised population groups, multidisciplinary teams, and implementation of a healthy lifestyle program with demonstrated success and translation. She oversaw the qualitative methods and analysis for this study.

Supplementary Table 2: Perceived potential barriers to recipient and family engagement in the Healthy Lifestyle Program

| **Perceived barrier to engagement** | **Example quote** |
| --- | --- |
| Conflict between recipients or families | *“If you put a whole lot of, um, people together from different nations or whatever, there's going to be cultural language barriers, uh, cultural um understandings, misunderstandings.”* – Table 1, Participant 4 (Male) |
| Busy lifestyle and timing of the program | *“Specific times when it's held, it might not be suitable for all.”* – Table 3, Participant 1 (Female) |
| Difficulties with family structure, or conflict within the family | *“Family issues in the home itself…family structure.”* – Table 1, Participant 5 (Male) |
| Financial and food insecurity | *“…maybe because they couldn't buy food for the family, it keeps them away because of the shame factor that comes into it.”* – Table 3, Participant 4 (Female) |
| Drug and alcohol use | *“I would say that some of the young ones, mothers, fathers, have different priorities, and in today's lifestyles and things, we must, we've got to take it on board that some of the young ones are into drugs and parents prioritise that as being more important than what this [the healthy lifestyle program] would be…”* – Table 4, Participant 3 (Female) |
| Intergenerational trauma and pre-existing mental health conditions | *“Also, a lot of the children these days, that would encompass our kids as well as our grandkids and great grandkids, they're suffering trauma from their parents…how they're going to snap out of things? They can't get to this program to help them out. They're going to have to come out of their shell, unna, somehow. You know what I mean?”* – Table 5, Participant 1 (Female) |
| Lack of motivation to engage (either parents or recipients) | *“No ‘get up and go’…no motivation to the parents of the children.”* – Table 2, Participant 2 (Female) |
| Accessibility of group sessions | *“Do they have a bus fare? If they don't have cars at home. Parents, grandparents, whatever, if they've got no cars…”* – Table 5, Participant 1 (Female) |
| Inappropriate venue location | *“The venue. A lot of our mob, there's certain venues they don't like going to.”* – Table 4, Participant 2 (Female) |

Supplementary Table 3: Participant-identified mitigation strategies for barriers to recipient engagement, and adaptations made to the Healthy Lifestyle Program

| **Barrier to engagement** | **Mitigation strategy** | **Program adaptation** | **Example quote** |
| --- | --- | --- | --- |
| Conflict between recipients or families; Difficulties with family structure, or conflict within the family | Conduct background cultural research before meeting families or forming groups | Initial community connector liaised with families prior to engagement with the program during inception | *“Plan your meet and greet. If you know you're going to meet someone, you got to research it, then go there.”* – Table 1, Participant 5 (Male) |
| Conflict between recipients or families | Develop ground rules with recipients for group sessions | A group agreement was developed in consultation with recipients and reinforced at each group session | *“But go back to the ground rules, needs to be made by everybody. The ground rules need to be made by all people who participate”* – Table 3, Participant 1 (Female) |
| Inappropriate venue location | Comfortable and familiar venue | Partnered with Child and Parent Centres based in schools (including Aboriginal community colleges) for conducting health assessments and schools for holding group program sessions | *“Venues I've just got to say, because this [the location of the workshop] is a familiar place for us now, that makes us feel safe, because it's a familiar environment.”* – Table 4, Participant 3 (Female) |
| Accessibility of group sessions; Busy lifestyle and timing of the program | Increase accessibility of sessions, including with home visits, rotating venues, adapting timing to other commitments etc | Home visits offered for health checks with flexible appointment times. Group sessions run after school. | *“Community centres, because it'll be a lot easier for us to go because we all know where the community centre is…Home visits as well.”* – Table 5, Participant 5 (Female) |
| Financial and food insecurity | Link families with free or reduced-cost food options | Program has become an approved referrer to Foodbank to provide food and grocery relief. Have also linked with KidSport to provide vouchers for financial assistance with sporting club fees. | *“Can we give, like, a food um box, what do you call it?...* *Foodbank, yeah, that's it. Foodbank.”* – Table 1, Participant 3 (Male) |
| Financial and food insecurity | Provide budget support and financial planning | Group program includes a session on budgeting | *“Budgeting support to enable budget planning/accounting.”* – Butchers’ paper notes, addressing transport barrier |
| Busy lifestyle and timing of the program | Help families establish routine and schedules | Sessions included on parenting and elements affecting wellbeing | *“Yeah, routine for family and kids, and so carrying that idea of routine”* – Whole group discussion (Male) |
| Accessibility of group sessions | Provide alternative transport options, either directly or by linking with other organisations | Partnered with Shire of Mundaring, and developed an established pathway to refer families to Moorditj Koort (Aboriginal Community Controlled Health organisation) and other groups for transport assistance | *“[Partner with] all the different agencies who do transport.”* – Table 1, Participant 3 (Male) |
| Busy lifestyle and timing of the program | Session reminders (SMS, email, mail, fridge brochures) and regular contact with families | Brochure with session content provided in advance. SMS reminders sent. Families phoned if indicated. | *“The time, like, a reminder. Um, text message reminder…* *And maybe get 'em to do their own little timetable. Stick it on the fridge.”* – Table 1, Participant 5 (Male) |
| Busy lifestyle and timing of the program | Sticking to schedule | Curriculum developed and calendar followed for group sessions | *“Whoever's presenting or taking the information needs to be considerate of people's time to make sure that they're finished on the same time. That timeframe needs to be kept to, the timeframe, and not go over.”* – Table 3, Participant 4 (Female) |
| Lack of motivation to engage (either parents or recipients) | Inspirational mentor to help increase motivation to engage | Community connector identified and seconded for program inception. Motivational interviewing and regular contact with families from program staff. Need for community champions identified | *“One would be, um, a mentor…someone to inspire them…For somebody who's been in ground zero for so long and then who then made it on their own, basically…”* – Table 1, Participant 4 (Male)  *“We need somebody, like, who does a lot in the community but not recognised for it…* *those sort of people will be inspirational to help motivate people.”* – Table 1, Participant 4 (Male) |
| Lack of motivation to engage (either parents or recipients) | Make program appear interesting and engaging, offer incentives | Incorporation of fun physical activities and healthy snacks at each group session. Sessions designed to be interactive and personal folders with program content developed | *“But also you've got to have an interesting program. No good using words. No good using words when you're talking to kids. You got to have symbols of everything…You get some kind of incentive at the end if you complete it.”* – Table 3, Participant 1 (Female) |
| Intergenerational trauma and pre-existing mental health conditions; Drug and alcohol use | Mental health support and building self-esteem | Screening tool for social emotional wellbeing included, with opportunity for psychologist review and referral for further assessment and support if indicated, following multidisciplinary team discussion | *“They need counselling. They need, like, intensive help.”* – Table 5, Participant 1 (Female) |
